# Research on Development and Challenges of Forest Food Resources from an Industrial Perspective—Alternative Protein Food Industry as an Example

**DOI:** 10.3390/foods14203503

**Published:** 2025-10-14

**Authors:** Yaohao Guo, Cancan Peng, Junjie Deng, Xiya Hong, Bo Zhou, Jiali Ren

**Affiliations:** 1 State Key Laboratory of Utilization of Woody Oil Resource, Central South University of Forestry and Technology, Changsha 410004, China; 20241200690@csuft.edu.cn (Y.G.); 20231200685@csuft.edu.cn (C.P.);; 2 Hunan Key Laboratory of Forestry Edible Sources Safety and Processing, Changsha 410004, China; 3 School of Food Science and Engineering, Central South University of Forestry and Technology, Changsha 410004, China

**Keywords:** forest food, protein resources, industrial development, alternative protein, challenges

## Abstract

The forest food industry, as a typical low-carbon green ecological industry, holds strategic significance in addressing global food security challenges. This review takes forest protein resources as an example to analyze the current development status, opportunities, and challenges from a global industrial perspective. Research indicates that forests, as a vital food treasure for humanity, can provide diverse protein sources such as insects, plants, microorganisms, and bio-manufactured proteins. Currently, numerous technological innovations and market practices have emerged in fields such as insect protein (e.g., there are over 3000 edible insect species globally, with a market size of approximately USD 3.2 billion in 2023, projected to reach USD 7.6 billion by 2028), plant-based alternative protein (e.g., plant-based chicken nuggets by Impossible Foods in the United States), microbial fermentation protein (e.g., the production capacity of Solar Foods’ production base in Finland is 160 tons per year), and cell-cultured meat (e.g., cell-cultured chicken is sold in Singapore), demonstrating significant potential in alleviating food supply pressures and reducing environmental burdens. However, industrial development still faces practical challenges including insufficient resource exploration, incomplete nutritional and safety evaluation systems, low consumer acceptance, high costs of core technologies (e.g., the first cell-cultured meat burger in 2013 cost over 1 million USD/lb, and current costs need to be reduced to 17–65 USD/kg to achieve market competitiveness), and imperfect regulatory mechanisms (e.g., varying national standards lead to high compliance costs for enterprises). In the future, it is necessary to achieve efficient development and sustainable utilization of forest protein resources by strengthening resource exploration, clarifying the basis of nutrients, promoting multi-technology integration and innovation, and establishing a sound market access system, thereby providing solutions for global food security and high-quality development of the food industry.

## 1. Introduction

Grain represents a fundamental pillar of global and national food security, playing a critical role alongside livestock, fisheries, and other essential commodities. In recent years, climate change and crop diseases have resulted in substantial grain yield losses [1], contributing to 735 million people globally experiencing malnutrition in 2022, representing 9.2% of the global population [2]. To meet the nutritional requirements of the projected global population of 10 billion by 2050, it is estimated that global food production must be increased by 50% over the next 25 years [3]. Accordingly, exploring multiple pathways for edible resource exploration and development, coupled with food innovation, has emerged as a pivotal strategy to address food security challenges. “World Resources Report: Creating a Sustainable Food Future” outlines five categories encompassing 22 potential solutions: (1) curbing the growth in demand for food and other agricultural products; (2) boosting food production without expanding agricultural land; (3) protecting and restoring natural ecosystems; (4) augmenting aquatic food supply; and (5) reducing agricultural carbon emissions [3]. To implement these solutions, robust exploration of diverse food resources, establishment of a diversified food supply system, and ensuring efficient and sustainable production and distribution of food are imperative. In recent years, global researchers have identified forests as a viable source of human food [4], animal feed [5], and essential food nutrients [6]. The emerging forest food industry holds the potential to exhibit attributes such as geographical accessibility, economic affordability, and sustainable management practices, though the extent of these advantages is highly dependent on regional contexts, specific ecosystems, and species [7]. Consequently, where these conditions are favorable, the industry could significantly enhance food supply security and alleviate malnutrition.

Forests may have functioned as a natural reservoir sustaining food resources for people living near forests—especially in communities with poor access to markets—thereby contributing to their dietary quality by providing the three essential macronutrients: carbohydrates, fats, and proteins [8]. According to the *China Forestry and Grassland Statistical Yearbook 2022*, the total output of edible forest products in China reached 22.425 million tons in 2022. Breakdowns included approximately 17.647 million tons of fruits, 1.281 million tons of dried fruits, 0.352 million tons of forest-derived beverages, 0.188 million tons of forest-based seasonings, and 801,400 tons of forest food (encompassing 473,000 tons of bamboo shoots, 231,000 tons of edible fungi, 40,200 tons of wild vegetables, 15,000 tons of Chinese toon, and 45,000 tons of other categories). Additionally, forest medicinal materials and woody oil crops accounted for 773,000 tons and 935,000 tons, respectively [9]. Presently, China’s annual production of forest food exceeds 200 million tons, with a per capita supply of approximately 130 kg, ranking among the highest globally and establishing forest food as the third most critical agricultural product after grains and vegetables. The report to the 20th National Congress of the Communist Party of China, alongside Central Document No. 1 of 2023 and 2024, explicitly stated the following: “Adopt a comprehensive perspective on agriculture and food systems, and source calories and protein from croplands, grasslands, forests, oceans, as well as from plants, animals, and microorganisms, to develop food resources through comprehensive and multi-dimensional approaches.” The global food system is undergoing a necessary transition towards greater sustainability and diversification. This shift requires innovative approaches to natural resource management, as exemplified by strategic frameworks like China’s “Broad Food Concept” and the emphasis on the “Four Functions of Forests.” These approaches highlight the potential of scientifically utilizing forest resources to enhance food supply capacity and diversify food sources. However, the global scientific understanding of forest food industrialization—particularly regarding scalable production, life cycle assessment, and market integration—remains underdeveloped. Examining these policies provides a valuable context for identifying and addressing these universal research gaps.

To address the global food security crisis and mitigate malnutrition, significant technological innovations have been achieved over the past few decades across the entire food production chain—spanning from the cultivation and breeding of food resources to post-harvest processing. Notable advancements encompass genetic engineering for enhancing crop stress tolerance [10], food preservation technologies for prolonging shelf life [11], development of functional packaging materials [12], precision in-line monitoring systems for improved food safety [13], establishment of traceability big data platforms [14], integration of artificial intelligence (AI) and blockchain technologies in food supply chain management [15], and tissue engineering-based alternative protein production [16]. However, harnessing these technological innovations to address global food security and human malnutrition challenges necessitates their translation into commercially viable and scalable applications—with particular emphasis on the development of forest food sources, with forest protein sources in particular, as sustainable alternatives to protein-dense animal-derived foods [17]. Furthermore, driven by diverse economic, cultural, and health-related considerations, consumers’ individualized preferences exert a direct impact on their selection of types and sources of alternative food products [18,19].

**Figure 1:**
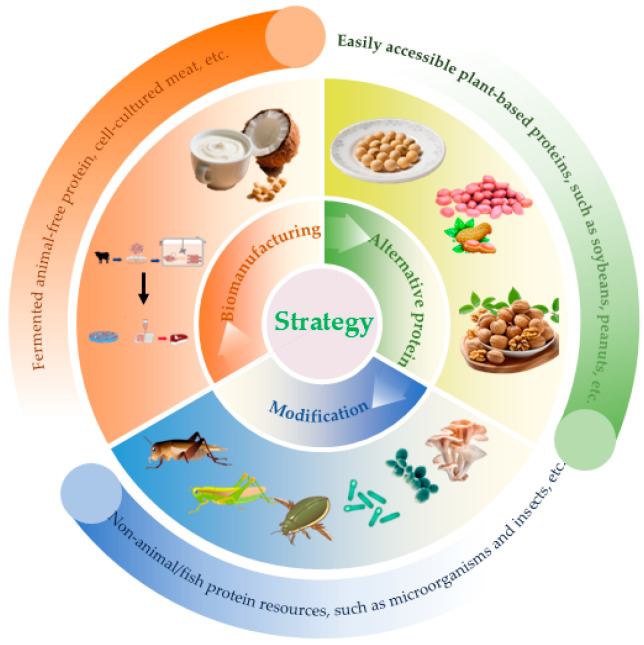
Strategies for finding forestry-derived protein.

To address the global protein demand driven by the projected population growth by 2050, the Food and Agriculture Organization (FAO) estimates that agricultural production must increase by 70%, with global meat and dairy output projected to rise by 4.35 million tons and 8.43 million tons, respectively [20,21]. Accordingly, identifying alternative sources with the capacity to produce high-quality protein under reduced temporal and spatial constraints has become imperative. Current scientific literature on protein food source development has primarily focused on production methodologies [22,23,24,25,26,27,28,29] and the exploration of specific alternatives to animal-derived proteins [26,27,28,29], including the utilization of protein substitutes, modification of existing non-animal and non-fish proteins, and bio-manufacturing of alternative proteins [30] (Figure 1). Among these avenues, forests represent a key reservoir for discovering alternative animal protein sources. However, a systematic review that synthesizes the technological innovation landscape from a holistic industrial perspective—encompassing scalability, economic viability, and consumer acceptance—is notably lacking. This review, focusing on forest protein food resources, systematically synthesizes the global development landscape of alternative livestock-derived protein food industries from a technological innovation perspective. It further proposes strategies to mitigate current challenges in the forest protein food sector, aiming to offer actionable insights for the high-quality development and modernization of the forest food industry.

## 2. Definition and Characteristics of Forest Food

### 2.1. Definition of Forest Food

In the international context, forest food is defined as animals, plants, and their derived products originating from forest ecosystems, which exhibit characteristics of original ecological integrity, non-contamination, healthfulness, and safety, and are suitable for direct or indirect human consumption [31]. These attributes are typically regarded as aspirational qualities rather than universally applicable criteria. The term is frequently conflated with “non-timber forest products” and is commonly linked to terminologies including “by-products” and “niche markets” [32].

In China, the definition of forest food encompasses two distinct categories: the broad sense and the narrow sense. In the broad sense, it is defined as all edible forest products derived from forest ecosystems, including plants, animals, microorganisms, and their processed derivatives, commonly referred to as “forest food products”. Rooted in the profound connotation of forests as “granaries”, the forest food concept emphasized by General Secretary Xi Jinping aligns strictly with the broad definition. In the narrow sense, the definition emphasizes adherence to sustainable forest management principles, specifically denoting various edible forest products (including their processed derivatives) that originate from pristine forest ecological environments, comply with relevant technical specifications, and exhibit attributes such as original ecological integrity, non-contamination, healthfulness, and safety [33].

### 2.2. Characteristics of Forest Foods

Within both domestic and international contexts, the definitions of forest food collectively converge to delineate its core characteristics: in terms of product scope, it is rooted in forest ecosystems, with edible forest products as the primary focus; in terms of origination environment, it is often associated with natural or relatively undisturbed forest ecosystems; in terms of technical frameworks, it is frequently expected to align with ecological principles of energy flow and material cycling, with limited or no reliance on chemically synthesized fertilizers and pesticides; in terms of product quality, it is aspirationally linked to internationally recognized standards of quality and safety. Collectively, in contrast to other food categories, forest food is characterized by five distinct attributes: ecological authenticity, edible safety, nutritional value, sustainability, and certification traceability.

## 3. Analysis of the Industry Status of Forest Food Protein Resources

The classification of forest food products is multifaceted: based on source, they are categorized into forest plant-based foods, forest animal-based foods, and forest microbial-based foods; based on primary nutritional components, they are further categorized into forest protein food resources, forest starch food resources, and forest oil food resources. This review specifically focuses on protein sources—including insects, plants, microorganisms, and large animals—to synthesize the current state of development and utilization of alternative proteins for livestock and poultry feed.

### 3.1. Analysis of the Industrial Status of Forest Insect Protein Resources

Edible insects boast a centuries-old history as a traditional food source worldwide [34]. Currently, it is estimated that 2 billion people globally consume insects as part of their diet, with over 3000 edible insect species identified and more than 1900 of these documented as food sources [35]—a practice predominantly observed in regions with high forest cover, such as Asia, Africa, South America, and Oceania. Boasting rich contents of proteins, essential amino acids, fatty acids, vitamins, dietary fiber, minerals, and other nutritional and functional components, edible insects have garnered increasing attention as a promising nutritionally healthy food source in Western developed nations in recent decades [36]. Although significant differences exist in nutritional composition between edible insects and conventional livestock meats—complicating direct comparisons of their nutritional value [37,38,39]—the protein content of edible insects (23.1–35.2 g/100 g) is typically higher than that of conventional livestock meats (19.2–21.5 g/100 g) [38]. In addition, the digestibility of insect proteins has been reported to range from 77% to 98%, further highlighting their potential as a sustainable alternative to animal-derived proteins [40].

According to a Rabobank report, the global demand for insect protein is projected to reach 500,000 tons by 2030, with the price per ton declining from EUR 3500–5500 during the scaling phase (i.e., 2020) to EUR 1500–2500 in the mature phase (i.e., 2030) [41]. Regarding market size, according to industry reports and trend forecasting models based on combined expert interviews and authoritative databases, the global edible insect market was valued at approximately USD 3.2 billion in 2023 and is projected to reach USD 7.6 billion by 2028, with a compound annual growth rate (CAGR) of 18.89% [42]. These estimates primarily refer to insect-based foods, although feed applications are also included. It should be noted that such projections carry inherent uncertainties, particularly with respect to regulatory approvals, consumer acceptance, and market penetration rates across different regions. Presently, activities of edible insect-based food startups are primarily focused on insect cultivation and rearing, as well as the development of food products and animal feed. Notable examples include Ÿnsect (Paris, France), which has developed a genotyping chip (Axiom®YNS_Mol1); Beta Hatch Inc. (Seattle, WA, USA), which utilizes gene-editing technologies (RNAi and CRISPR) to precisely engineer high-protein insects; and Hey Planet (Copenhagen, Denmark) together with YumBug (London, UK), which have developed insect-based protein bars, chocolates, and processed beetle meat products tailored to align with Western dietary preferences (Table 1).

The rapid growth of the edible insect industry is driven by key factors including resource accessibility and sustainability—such as low greenhouse gas emissions, minimal water and space requirements, and high feed conversion efficiency [42]—and, crucially, by multi-source financial investments from Western developed countries [43]. Despite the current highly fragmented nature of the edible insect market, it has garnered over USD 1.3 billion in cumulative investments in recent years [44]. For instance, Protirax and Ÿnsect have partnered with Tyson Foods and Pure Ultra Simple LLS to develop insect protein-based food products and animal feed (Table 1). Furthermore, governments across various countries have facilitated industry development through targeted initiatives: the Australian Centre for International Agricultural Research (ACIAR) and the International Centre of Insect Physiology and Ecology (ICIPE) jointly established the Emerging Insect Technology Hub (EIT-Hub) to develop technologies for insect protein-based food, feed, and fertilizer production [45]; the UK government allocated USD 7.5 million to Entocycle via the Industrial Strategy Challenge Fund (ISCF) to construct large-scale industrial edible insect farms utilizing food waste as feed stock [46].

### 3.2. Analysis of the Industrial Status of Forest Plant Protein Resources

Humans have a millennia-old history of consuming plant-based protein foods; for instance, soybeans were documented as a food source as early as the Han Dynasty in China [47]. Currently, commonly utilized protein-rich plants encompass legumes under large-scale cultivation (e.g., soybeans, mung beans, adzuki beans, chickpeas, peas, edamame, among others) [48], nuts and seeds (e.g., peanuts, almonds, cashews, walnuts, sunflower seeds, pumpkin seeds), cereals (e.g., wheat, quinoa, oats, coix seed), and certain algae (e.g., laver and spirulina). As a key strategy for mitigating environmental and food security challenges, the development of production technologies for diversified, high-quality plant protein products with growing consumer interest and gradual market acceptance has emerged as a central focus within academic and industrial sectors. Notable innovations include plant-based meat analogs/substitutes (PBMAs) and plant-based dairy analogs/substitutes (PBDAs) [26], with the goal of enhancing the texture, flavor, and sensory properties of PBMAs and PBDAs to match those of animal-derived meats (Table 1). Representative cases include Spain’s Novameat, which utilizes 3D bioprinting technology to fabricate fibrous and microfibrous structures, thereby improving the textural properties of PBMAs; Israel’s MeatTheEnd, which optimizes pretreatment technologies integrated with extrusion processes to produce textured plant proteins, enhancing the sensory profiles of PBMA products; and the United States’ Lipid, which employs encapsulation technology to develop animal fat-mimetic plant-based lipids, addressing sensory and nutritional limitations in PBMA formulations.

Based on the 2024 update of the Alternative Protein Company Database by Josh Bisig, Jennah Brown, and colleagues, over 1000 companies worldwide are currently involved in the production and marketing of plant-based protein foods, of which approximately 40% specialize in the research, development, and manufacturing of plant-based meat analogs (PBMAs) and plant-based dairy analogs (PBDAs) [49]. Notable examples include Kraft Heinz and Mondelez International’s non-dairy cream cheese products, Kellogg’s plant-based chicken waffle egg sandwiches, and Burger King’s plant-based offerings, as reported in “Plant-based Meat, Seafood, Eggs, Dairy, and Ingredients” published by the Good Food Institute (GFI) in 2024 [50]. In the United States, for example, the top three food companies—Kellogg, Maple Leaf Foods, and Conagra Brands—account for nearly 70% of PBMA sales, with Kellogg alone accounting for nearly 50% of the total market share [51].

The plant-based protein alternative market is exhibiting steady growth, driven by the inherent attributes of plant-derived proteins—including health-promoting properties [52,53], environmental sustainability [54,55,56], cost-effectiveness [57,58,59], and high consumer acceptability [60]. Notably, the global market value of plant-based dairy analogs/substitutes (PBDAs) is projected to grow from USD 25.19 billion in 2022 to USD 69.8 billion by 2030 [48]. In 2022, the number of distinct investors in plant-based protein food enterprises rose by 17%, totaling over 1500 [50]. Within the corporate sector, notable examples include Impossible Foods (Redwood City, CA, USA), which raised USD 500 million in funding, and Rebellyous, which secured USD 6 million to support R&D efforts for plant-based protein food development. At the governmental level, strategic investments further underscore this growth: the German government has allocated USD 41 million to support plant-based protein food innovation [61]; Denmark, Sweden, and Switzerland have collectively committed over USD 150 million to fund plant-based protein R&D [50]; and the Singaporean government has established the world’s first hybrid innovation center dedicated to plant cultivation and plant-based meat analog development [62].

### 3.3. Analysis of the Industrial Status of Forest Microbial Protein Resources

An additional source of alternative proteins encompasses microorganisms, including bacteria and fungi—with particular emphasis on yeast [63], molds, and macrofungi (edible fungi). Edible microorganisms, characterized by high protein content (50–85%), excellent bioactive properties, low fat and sodium content, as well as desirable textural attributes (e.g., chewiness) and meat-like flavor profiles [64], exhibit significant potential as non-meat protein substitutes. Microbial protein has long been a focus of interest within academic and industrial sectors owing to inherent advantages, including high production efficiency, broad substrate versatility, and facile scalability for industrial production. As early as the mid-1980s, the global annual output of microbial protein had already reached 2.0 × 10^6^ tons. According to projections by the Boston Consulting Group (BCG), the alternative protein market is expected to reach USD 290 billion by 2035, with microbial fermentation-derived proteins accounting for 22% of the total market share. Building on the theoretical potential of fungi to replace wood-based raw materials, substituting 20% of global beef consumption with microbial protein by 2050 could reduce the annual deforestation rate by approximately 56% and correspondingly lower carbon emissions. However, the extent to which these environmental benefits materialize depends heavily on the efficiency of waste material collection and the scalability of industrial-level production. Notably, when 1000 kg of substrate is cultured over a 24 h period, the protein yields are approximately 2000 kg for yeast, 10 kg for soybeans, and 1 kg for cattle, while the average protein content of microbial protein reaches 2–2.5 times that of meat and 1.7 times that of soybeans. More significantly, fungal protein produced through waste-based deep fermentation demonstrates substantial cost advantages—this technique utilizes low-cost residual materials as substrates, requires production cycles of only 3–60 days, and im-proves land use efficiency by more than tenfold compared to conventional agriculture. By integrating waste valorization with carbon emission reduction, it achieves a negative environmental footprint. Consequently, the production cost can be reduced to 1.6–5.5 USD/kg, significantly lower than that of animal-derived proteins (10–33 USD/kg) and certain plant-based proteins. These findings collectively validate the superior economic viability of microbial protein over animal- and plant-based protein sources across multiple dimensions, including production efficiency, resource utilization, and cost structure.

Driven by advances in modern food processing technologies, an expanding array of diversified microbial protein-based foods has been developed. Notable examples include Austria’s Revo Foods, which has leveraged extrusion and 3D printing technologies to produce fibrin-like matrices from filamentous fungi, yielding mycelium-based salmon fillets [65]; Sweden’s Mycorena, which employs liquid fermentation to generate fungal proteins and lipids, enhancing the sensory profiles of plant-based meat analogs (PBMAs) [66]; and a collaboration between Republic of Korea’s INTAKE Corporation and Israel’s Oshi Company, which utilizes high-density fermentation to process seaweed, resulting in seaweed protein-derived salmon and tuna alternatives. In academic research, Keerthana et al. prepared edible mushroom protein-based meat analogs with significantly improved physicochemical and sensory attributes [67], while Cho [68] and Mohamad Mazlan [69] separately employed twin-screw and single-screw extrusion to produce oyster mushroom protein-based meat analogs, achieving enhanced textural properties and antioxidant capacities. Commercially, the UK-based company Quorn ferments *Fusarium venenatum* to manufacture premium protein products characterized by high fiber content and low saturated fat.

For instance, the Ministry of Oceans and Fisheries of Republic of Korea has initiated a funding program commencing in May 2024, scheduled to run through 2028. A total of KRW 6 billion (approximately USD 4.3 million) has been allocated to support INTAKE Co. in scaling up the fermentative production of microalgae-derived blue protein. In Finland, Solar Foods has raised approximately USD 47 million in equity and aims to secure an additional USD 32 million in debt financing to support its public listing. Additionally, the company has received a USD 36.2 million grant under the European Important Projects of Common European Interest (IPCEI) to facilitate the development of industrial-scale microbial protein production. Further strategic initiatives include securing an EUR 76 million (USD 81 million) grant to expand production capacity. In Israel, Remilk and Imagindairy have secured venture capital investments of USD 120 million (2022) and USD 13 million (2021), respectively, to advance the fermentation-based production of animal-free whey protein analogs.

### 3.4. Analysis of Industry Status of Forest Animal Protein Resources (Biomanufactured Protein)

Biofabricated protein facilitates the production of animal protein by circumventing the feeding and slaughtering processes inherent to traditional animal husbandry, with its core technology rooted in multidisciplinary integrated cell culture techniques [27]. As a future food category, the production process of biofabricated protein and its derivative product—cell-cultured meat—involves four primary steps: (1) isolating target cells from animal tissues as “seed cells” for biofabricated protein cultivation and expansion; (2) mimicking the in vivo growth microenvironment to enable precise cell fermentation for exponential proliferation; (3) constructing the tissue architecture of cell-cultured meat using biological scaffolds or 3D bioprinting technology; and (4) restoring meat-like taste and texture through food science interventions to yield final meat products. The industrialization of cell-cultured meat typically encompasses four stages: laboratory-scale, pilot-scale, demonstration-scale, and commercial (industrial)-scale production. Two key technologies underpin the large-scale commercial production of biofabricated protein and its derivative cell-cultured meat. The first critical technology is precision fermentation. Driven by advancements in genomic technologies, precision fermentation—utilizing microbial hosts as “cell factories” to produce specific functional components—has enabled the synthesis of animal proteins, such as the heme protein used in plant-based meat burgers. According to a report by the U.S. Good Food Institute (GFI), there are currently at least 68 global companies employing fermentation technology to produce or support the development of animal-free alternatives to meat, eggs, or dairy products, with 44 focusing on novel protein fermentation applications and 24 on new protein industry product lines (Table 1). Representative cases include Perfect Day (Aliso Viejo, CA, USA) and Eden Brew (Pymble, NSW, Australia), which produce milk-derived proteins via precision fermentation to develop synthetic milk with taste, sensory attributes, and appearance comparable to animal-derived milk; Remilk and Imagindairy (Tel Aviv, Israel), which synthesize milk whey protein and casein through precision fermentation; Air Protein (Berkeley, CA, USA), which produces multifunctional protein-rich flour (with an amino acid profile analogous to meat protein) via precision fermentation; and Joes Future Food (Nanjing, Jiangsu, China), which prepares cell-cultured meat through muscle stem cell cultivation. The second key technology is 3D bioprinting, which integrates materials science with computer-aided design (CAD) model data to fabricate three-dimensional structures [70]. Scientists at Osaka University (Suita, Osaka, Japan) have utilized 3D bioprinting to replicate the complex architecture of muscle fibers, fat, and blood vessels, yielding wagyu beef with characteristics similar to native beef [71]; Mooji Meats (Cambridge, MA, USA) has developed a 3D bioprinting platform that replaces conventional texturing techniques. Additionally, the research team led by Academician Changhu Xue at Ocean University of China successfully constructed an edible 3D porous scaffold based on pea protein by combining Pickering emulsion gel templating and double cross-linking technology [72].

Biofabricated protein (i.e., cell-cultured meat) has taken just over 20 years to progress from conceptualization to commercial product launch. The world’s first cell-cultured meat company, Memphis Meats (subsequently renamed Upside Foods in 2021), was founded in the United States in 2015. By 2022, there were 156 companies globally dedicated to the research and development of biofabricated protein and cell-cultured meat, with 27 facilities scaling to pilot scale or beyond (Table 1). These include chicken producers (e.g., Upside Foods and GoodMeat Emeryville, CA, USA/Eat Just, Alameda, CA, USA), beef producers (e.g., Aleph Farms, Rehovot, Israel), pork producers (e.g., Meatable, Leiden, Netherlands; Joes Future Food, Nanjing, Jiangsu, China), seafood producers (e.g., Wildtype, San Francisco, CA, USA; Avant Meat, Hong Kong Science Park, Hong Kong China), and foie gras producers (e.g., Gourmey, Paris, France). Notably, in April 2024, the Finnish company Solar Foods established the world’s first large-scale air protein production facility in Helsinki, with an annual production capacity of up to 160 tons—equivalent to the protein output of a 300-cow cattle farm [73].

According to life cycle assessment (LCA) analyses, compared with conventional livestock production, the production of biosynthetic proteins (i.e., cultured meat) can reduce energy consumption by 7–45%, water use by 82–96%, greenhouse gas emissions by 78–96%, and land occupation by up to 99%. However, current LCA studies are constrained by the limited technological maturity and data availability, and further verification of its sustainability requires large-scale production and standardized evaluation [17]. Currently, capital investment in this sector continues to expand, driven by diverse funding sources including 679 independent investors, at least 35 major institutional partners, and public sector entities, with cumulative investments reaching USD 2.8 billion [17]. Notable corporate financing cases include Memphis Meats—the pioneering cell-cultured meat company—which had secured cumulative financing of USD 161 million by 2021; Aleph Farms, which raised USD 140 million to advance the development of animal-free biofabricated proteins and their derived food products; and Vow, which secured nearly USD 60 million for biofabricated protein research (Table 1). At the governmental level, the UK government has allocated GBP 2 billion to support the development and manufacturing of alternative proteins, specifically including cultured meat [74]. In September 2022, the U.S. Biden administration launched the “Bioeconomy” initiative, explicitly announcing financial support for “foods derived from cultured animal cells.” Market data indicates that in 2019, fermentation protein companies in the U.S. secured over 3.5 times more funding than all biofabricated protein (cell-cultured meat) companies, while accounting for nearly 60% of total financing in plant protein companies. Even in 2020, fermentation protein companies raised a total of USD 837.25 million, with examples including MycoTechnology’s USD 39 million Series D funding and Perfect Day’s USD 300 million Series C funding.

## 4. Challenges

**Figure 2:**
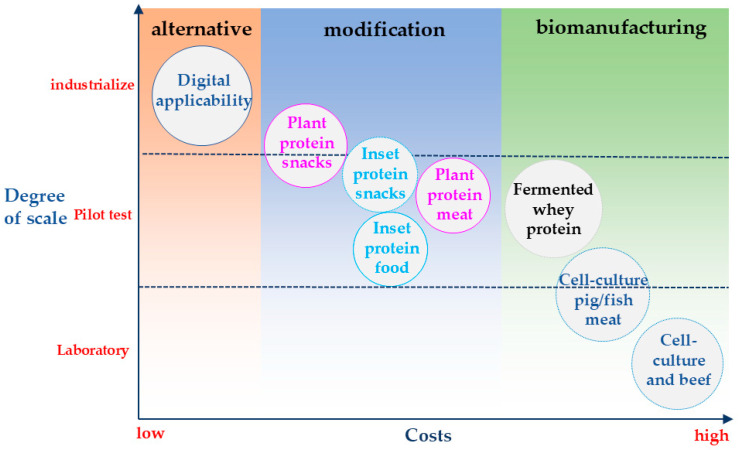
Analysis of market penetration potential based on cost and scalability for forestry-derived protein foods.

**Figure 3:**
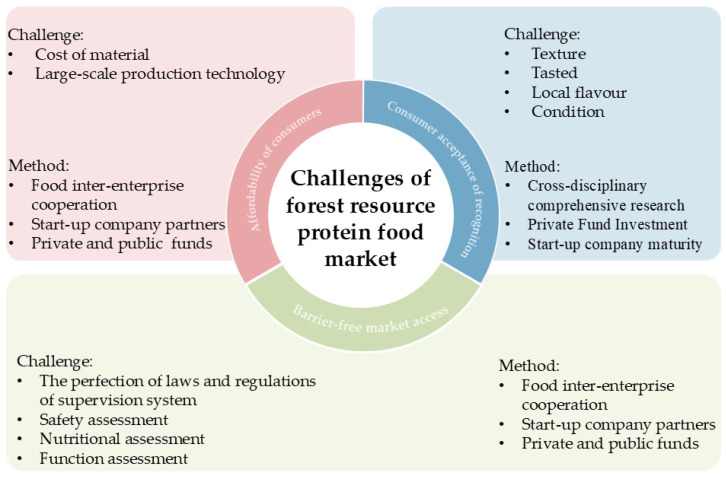
Challenges and approaches for mass market of forestry-derived protein foods.

In summary, to address the global food supply–demand imbalance driven by climate change and population growth, the food industry has proactively pursued alternative protein sources, innovated food production technologies, and accelerated the translation of groundbreaking research into commercial applications over the past two decades, yielding substantial advancements. Notably, food and meat analogs derived from forest plants, insects, and forest fungi have penetrated supermarket shelves and restaurant menus, while several alternative protein products have gradually garnered preliminary regulatory approval across multiple countries. For example, in northern and northeastern Thailand, fried bamboo caterpillars and crickets are widely available in markets and restaurants as popular delicacies. In Papua, sago grubs are directly sold in markets and serve as one of the most important local protein sources. In the United States, fried crickets and silkworm pupae are often offered during educational activities to introduce edible insects to the public [75]. However, to achieve large-scale popularization of such products, numerous practical challenges must still be overcome. First, the practical economic viability of large-scale production remains a subject of significant debate; particularly against the backdrop of current high energy prices, energy consumption costs may emerge as a critical bottleneck restricting its commercialization (Figure 2). Second, regarding consumer acceptance, existing survey findings cannot be readily extrapolated to broader populations, especially consumer groups with deeply rooted traditional dietary beliefs (Figure 3). Furthermore, the complexity of “market access regulation” also poses a significant challenge: variations in regulatory standards across countries not only substantially increase corporate compliance costs but also reflect the fact that regulatory authorities have yet to reach a consensus on the potential risks of such novel foods (e.g., allergens, long-term health effects, etc.) (Figure 3). Thus, current so-called “technological breakthroughs” are largely confined to the laboratory scale, and the realization of commercial implementation and social acceptance is far from being achievable through a single technological breakthrough alone; rather, it represents a systemic challenge involving economic, cultural, policy, and consumer psychological dimensions.

### 4.1. Resource Mining on Fortified Plant Protein, Deepened Microbial and Insect Protein, and Reserve Animal Protein

Global research, development, and utilization of plant-based proteins have yielded substantial advancements. However, relative to the vast plant resources in forest ecosystems—with a conservative estimate of 500–1000 economic forest tree species yielding forest food in China [48]—their development and utilization rates remain suboptimal, underscoring the urgent need to further harness the potential of forest plant protein resources. Concurrently, efforts should be directed toward enhancing technological innovation in the development and utilization of legume, cereal, and nut proteins to improve the commercial viability and palatability of plant protein-based foods by optimizing their quality, flavor, and sensory profiles.

Over 3000 edible insect species have been identified globally, with over 1900 species documented as food sources [35]. These include beetles from the family Scarabaeidae (e.g., *Tenebrio molitor*, *Alphitobius diaperinus*, and *Zophobas morio*); Orthoptera (e.g., *Acheta domesticus*, *Gryllus bimaculatus*, *Locusta migratoria*); and Lepidoptera (e.g., *Galleria* spp. and *Silkworms*). In China, 324 edible or entomophagy-relevant insect species spanning 11 orders have been recorded, with distributions as follows: Lepidoptera (37.65%), Coleoptera (18.21%), Hymenoptera (15.43%), Orthoptera (13.27%), Hemiptera (6.17%), Isoptera (4.94%), Uropygi (1.54%), and remaining orders (2.79%, encompassing Megaloptera, Ephemeroptera, Diptera, and Blattodea). However, only 10–20 species are commonly consumed [76].

Microbial proteins, particularly edible fungal proteins, boast a millennia-old history of human consumption, dating back to China’s Yangshao period (6000–4000 BCE). Currently, over 2000 edible microbial species have been identified globally; however, only approximately 25 are utilized as food sources, with a small subset undergoing commercialization [77]. Globally, the most widely consumed microbial species are the fungi *Agaricus bisporus* and *Pleurotus* spp. [78], with the highest production volumes of edible fungi concentrated in China, the United States, the Netherlands, Poland, Spain, and France [79]. Microbial proteins are currently primarily derived from yeast, spirulina, microalgae, and mushrooms (e.g., oyster, shiitake, and enokitake mushrooms). Among these sources, species such as white enokitake, shiitake, portobello, chanterelles, and enokitake have been explored as potential substitutes for animal-derived products including beef, crab meat, and chicken, although their use and acceptance vary across regions and applications [80].

China boasts abundant wildlife resources, with over 4400 vertebrate species alone, accounting for more than 10% of the global total. Currently, animal cells utilized in precision fermentation for biofabricated protein production—specifically for cell-cultured meat—are primarily derived from muscle stem cells and oocytes of domestic pigs, cattle, chickens, and salmon. Notably, limited research has been conducted on isolating target muscle stem cells from forest-dwelling animals for biofabricated protein and cell-cultured meat development. For example, in 2023, the Australian company Vow announced the successful biofabrication of a mammoth meatball using DNA from extinct mammoths.

In summary, priority should be given to strengthening the exploration and utilization of high-value forest plant protein resources, with a focus on expanding the development of forest insect and microbial protein resources. High-throughput screening and characterization tools should be employed to identify promising microorganisms exhibiting high fiber content, low saturated fat levels, and high-quality protein profiles.

### 4.2. Strengthen the Functional, Nutritional, and Safety Assessment of Protein Food Resources, Clarifying the Basis of Edible Substances

Globally, the nutritional value of approximately 200 species of edible, feed, and medicinal insects has been evaluated. These studies have consistently demonstrated that edible insects are rich in protein (≥50%) and contain a complete profile of 18 amino acids. Notably, their content of eight essential amino acids required by humans is 2-10 times higher than that found in meat, eggs, and dairy products [81]. Substantial research has been conducted on the nutritional and functional assessment of microbial proteins (19-35%), particularly edible fungi. These studies have investigated their pharmaceutical activities (including antitumor, antibacterial, and antiproliferative effects) [64], flavor-enhancing and nutritional contributions [82], and regulatory effects on gut-related diseases [83]. Collectively, this research confirms that microbial proteins can serve as a stable resource for developing nutritional supplements and functional foods [84]. Given that the bioavailability of plant- and microbial-based proteins is generally lower than that of animal proteins, greater emphasis should be placed on the actual bioavailability of nutrients during the development and utilization of forest plant and microbial proteins. This approach is essential for determining whether proteins and their processed products derived from plant and microbial resources represent healthier alternatives [85,86,87,88]. Currently, the nutritional and functional evaluation of biofabricated proteins (e.g., cell-cultured meat) remains underexplored, with greater emphasis placed on textural attributes and sensory properties—such as differences in cytoskeletal proteins between cultured and conventional meat [89]. From a safety standpoint, forest protein resources pose potential hazards, including biological contaminants (e.g., bacteria, viruses, fungi, parasites), chemical pollutants (e.g., pesticides, heavy metals, flame retardants), as well as potential allergic reactions (particularly to insect-derived and biofabricated proteins). A food poisoning outbreak in Thailand showed that unrefrigerated fried insects could accumulate histamine at hazardous levels, causing severe allergic reactions. Similarly, studies have reported arsenic accumulation in black soldier fly larvae and strong cross-reactivity in shrimp-allergic patients consuming mealworms, both indicating high safety risks. [90,91]. Thus, comprehensive research and monitoring are needed to assess their quality and safety profile [90], facilitating the efficient identification and safe mitigation of risks. Furthermore, anti-nutritional factors in forest protein resources deserve attention: notably, high nucleic acid content (4–18%) in microbial proteins may induce gout in susceptible populations, while phenolic compounds in plant-based proteins can lead to nutritional antagonism.

Numerous studies have indicated that the introduction of any new food raw material, food-related variety, or novel food additive necessitates addressing key challenges such as consumer acceptability and safety assessment [92]. Currently, the material basis and characterization of forest protein food resource products remain insufficiently explored, specifically including the structural properties of major functional components, risk factors, anti-nutritional factors, as well as their bioavailability and optimal intake levels. This gap thereby results in insufficient precision in quality control protocols during production processes and low efficiency in converting resources into high-value, high-quality food products, ultimately restricting the achievement of full-component utilization, multi-scale large-scale manufacturing, and industrial implementation of forest food resources. From a nutritional perspective, although the protein content of certain forest protein resources is lower than that of conventional livestock-derived meats (e.g., pork and beef) [92], they exhibit rich profiles of nutritionally active components, including dietary fiber, bioactive peptides, terpenes, and carbohydrates. These resources also exhibit lower levels of energy, saturated fat, sodium, and cholesterol, and higher dietary fiber content, which may offer potential health advantages when compared with animal-derived meats [93]. Looking forward, efforts should focus on continuously innovating and developing advanced detection and processing technologies, as well as enhancing analytical investigations into the nutritional and functional components of forest-sourced proteins and potential risk factors associated with food production (e.g., biological and chemical contaminants), to enhance food quality and safety [94]. Additionally, it is critical to conduct in-depth analyses of the mechanisms through which the abundance or deficiency of various nutrients and metabolites in forest protein resources impact consumers’ short-term and long-term health outcomes. Such research will provide a theoretical foundation for their scientific utilization and industrial advancement.

### 4.3. Research and Creation of Consumption-Oriented Food Innovation and Intelligent Manufacturing Technology

Contemporary studies suggest that consumer acceptance of forest-derived protein products—particularly insect proteins and animal-derived cell-cultured proteins—is generally limited. For example, a nationwide survey of 1830 consumers across the four major regions of the United States found that 72% still preferred conventional beef, while only 28% chose alternative proteins, with plant-based meat accounting for approximately 16% and cultured meat only 5%. Willingness to pay for cultured meat (USD 2.18–4.28/lb) was substantially lower than for plant-based meat (USD 5.04–6.30/lb), and interventions related to branding or information had limited impact on market share. Acceptance varied across demo-graphic groups, being higher among vegetarians, males, younger individuals, and those with higher education and lower among consumers over 45 years old and those favoring conventional beef. These findings further indicate that overall consumer acceptance of alternative proteins in the United States remains limited. Research has demonstrated that purchase intention toward such products is primarily influenced by personal motivations, food preferences, and sensory experiences [95]. From an industrial standpoint, technological innovation is necessitated to develop market-appealing products and enhance consumer acceptance, with core strategies encompassing the optimization of sensory attributes (e.g., taste, flavor, and texture) and reduction of production costs [96]. Such technologies include green and efficient purification and preparation processes for forest-derived protein resources, high-density precision fermentation, innovative formulation optimization, and texturization technologies (e.g., extrusion and 3D printing).

Significant advancements have been made in the development and utilization of plant protein resources, including via breeding improvement and nutritional fortification to mitigate nutritional imbalances in raw materials [97]. To alleviate off-flavors in plant-based legume beverages, endogenous regulation (e.g., breeding, germination) and exogenous regulation (e.g., inhibition of relevant enzyme activities, fermentation-based treatments) have been employed [98]; furthermore, partial hydrolysis, chemical modification, and high-pressure and ultra-high-pressure homogenization have been utilized to enhance the physical stability of legume beverages [60], in which the unique advantage of ultra-high-pressure homogenization lies in its capacity to concurrently tackle the three primary stability issues: physical stability (sedimentation and stratification), chemical stability (oxidative rancidity), and microbial stability (shelf life). Conversely, product development for forest insect protein resources necessitates innovation via emerging food processing technologies to develop diverse food matrices with strong consumer acceptance, including enhancing the solubility, emulsifying properties, and other functional attributes of high-foaming edible insect proteins [99,100].

One of the key barriers hindering the industrialization of biofabricated protein (cell-cultured meat) is the limitation of cell proliferation in 2D culture systems, where laboratory-scale cell-cultured meat is typically constrained to millimeter-scale dimensions—far below commercial requirements. Although studies have reported the successful expansion of crucian carp skeletal muscle cells using fish gelatin-based porous microcarriers and the bioassembly of centimeter-scale cell-cultured fish meat [100], marking a significant technological advancement in this field, a critical examination from an industrialization perspective reveals notable limitations in the current system. First, the existing cell expansion efficiency remains insufficient to meet the demands of large-scale production. Second, the low reproducibility of such sophisticated bioassembly processes in industrial-scale bioreactors may lead to poor product consistency and increased manufacturing costs. These key technical bottlenecks, coupled with high medium costs and substantial process energy consumption, collectively constitute the fundamental reasons for the persistently high production costs of cell-cultured fish meat. Therefore, future research should not be limited to laboratory-scale feasibility verification but should focus on promoting substantial breakthroughs in core aspects such as large-scale manufacturing and cost control. For example, the production cost of the world’s first cell-cultured meat hamburger exceeded 1 million USD/Ib in 2013; Wildtype’s sushi-grade cell-cultured salmon incurred a production cost of 1.18 USD/g (Table 1); and Nanjing Zhouzi Future Food Technology Co., Ltd. initially projected the price of cell-cultured pork belly to be several hundred yuan per kilogram at launch. Notably, projections indicate that cell-cultured meat production costs must be reduced to 17–65 USD/kg to achieve market competitiveness [17,101]. Therefore, the key challenge in advancing cultured meat from millimeter-scale laboratory samples to commercial production lies in the systemic transition from two-dimensional (2D) planar culture to three-dimensional (3D) culture.

The second recognized challenge in the industrialization of cell-cultured meat is that the product still consists primarily of cellular biomass, with notable differences in texture, mouthfeel, nutritional composition, and flavor compared to conventional meat. Although technologies including plant protein scaffolds, 3D bioprinting, and electrospinning have been employed to enhance the consumer acceptance and quality of cell-cultured meat, breakthroughs in decimeter- to meter-scale large-scale culture technologies, as well as precise modulation of texture, mouthfeel, and nutritional flavor, remain pressing challenges for the food science and industry sectors. Currently, innovative research on sensory experience enhancement technologies is primarily focused on plant-based protein products. Despite advancements in biofabricated protein (cell-cultured meat) technologies over the past decade—encompassing protein extraction, large-scale microbial precision fermentation, and food formulation—which have yielded progress in improving sensory and flavor attributes (Table 1), research in this domain remains limited. Notable examples include Gourmey’s improvement of the delicate flavor and creamy texture of cultured foie gras via compound blending and Wildtype’s production of sushi-grade cultured salmon using salmon oocytes. Recent studies have demonstrated that gelatin-based hydrogel functional scaffolds can simulate the cooking properties of traditional meat and enhance the aroma and sensory quality of cultured meat [102]. Additionally, the selection of non-allergenic cell scaffold materials is critical for supporting cultured cell adhesion, proliferation, and differentiation, as well as for maintaining the three-dimensional architecture of cell-cultured meat [103].

Innovative research on product shaping technologies focuses on advancing scaffold construction strategies to simulate meat-like morphological features, with key approaches including extrusion and 3D bioprinting. Among these technologies, extrusion technology has emerged as the mainstream shaping method in current protein-based food manufacturing, owing to its high production efficiency, cost-effectiveness, and superior energy efficiency [104]. Food 3D [70] and 4D [105] bioprinting technologies enable precise modulation of food shape, color, flavor, nutritional composition, and temporal dynamics at the laboratory scale, offering potential for functional property enhancement and personalized food customization. Future 3D food printing should advance toward multi-material systems, with a focus on exploring broadly applicable correlations between rheological properties, printing parameters, and resultant product performance [101]. Hybrid integration of extrusion and 3D printing has emerged as a novel trend. For instance, the South Korean company INTAKE employs a six-axis robotic arm to conduct lamination and gelation processes on seaweed, subsequently utilizing 3D printing technology to achieve large-scale replication of the taste, texture, aroma, and color profiles of salmon and tuna, thereby developing seaweed-based alternatives to these seafood products [106].

In the context of future development and utilization of forest protein resources, the integration of 3T technologies (modern Food Technology, Biotechnology, and Artificial Intelligence technology) is poised to serve as a fundamental pathway for addressing a range of core challenges, including key technical issues and engineering cost constraints in this industrial sector. For instance, the cost of cultured meat produced by the American company SciFi Foods using CRISPR technology has been dramatically reduced compared to the cultured meat burgers developed by Mark Post’s team and Peter Verstrate’s company a decade ago (Table 1). From a technological perspective, however, the development of forest protein resources still faces multiple hurdles, including allergenicity risks, excessive seasoning addition for flavor enhancement, inefficient lipid–protein interface fusion, microbiological safety concerns under high-humidity, neutral-pH storage conditions, and challenges in controlling product stability and off-flavor formation [107]. These factors collectively restrict the large-scale application and cost reduction of forest proteins. Therefore, efforts should focus on achieving collaborative innovation of 3T technologies through interdisciplinary integration to facilitate the efficient and high-value utilization of forest protein resources. Concurrently, based on the inherent properties of protein resources, product formulations, and processing characteristics, comprehensive disclosure of protein sources and nutritional composition information to consumers should be implemented to enable informed decision-making aligned with individual needs.

### 4.4. Building a Perfect Market Access Supervision System

As a category of “three new foods,” the achievement of full commercialization for forest resource protein foods necessitates overcoming not only technical bottlenecks but also navigating regulatory and administrative barriers, such as the absence of standardized regulatory frameworks, streamlined administrative approval processes for market entry, and established food certification systems [29]. In particular, the development of comprehensive regulatory frameworks and risk assessment protocols—encompassing biological and chemical hazards—for forest resource protein foods requires urgent strengthening. The current regulatory landscape significantly prolongs the time-to-market for cell-cultured meat, as it requires navigating complex multi-agency approvals—such as novel food assessments in the EU or joint FDA–USDA reviews in the U.S.—amid evolving technical standards. Labeling presents another critical hurdle, as conventional definitions of “meat” do not apply, necessitating clear disclosure of product origin and potential genetic modifications. Furthermore, comprehensive safety testing is mandated across the entire production chain, encompassing screening for pathogens in cell sources, monitoring residues in culture media, assessing scaffold material safety, and controlling migration substances from novel processing technologies like 3D bioprinting. While these multifaceted regulatory requirements help ensure safety and compliance, they collectively pose significant challenges to market entry. Currently, no globally harmonized safety governance standards exist for hazard mitigation in forest resource protein foods; however, countries worldwide have proactively launched exploratory initiatives. For instance, regulatory authorities in the United States, Canada, and other jurisdictions are endeavoring to develop safety assessment frameworks and regulatory systems for cell-cultured meat, with emphasis on mitigating exogenous chemical contaminants during production and endogenous biological hazards in animal cell cultures. The EU Novel Food Regulation formally categorized cell-cultured meat as a novel food as early as 2018. In 2022, the Chinese government outlined in its 14th Five-Year Plan for Bioeconomy Development strategies to advance synthetic biology technologies, investigate novel foods such as “synthetic proteins,” drive iterative advancements in the food industry, and mitigate environmental and resource constraints associated with traditional animal husbandry.

Exploring forest resource proteins and producing related foods have garnered recognition from governments and non-governmental organizations as critical to advancing high-quality national economic growth, environmental sustainability, and innovative food production [17]. Globally, governments are actively developing and refining regulatory frameworks and legal systems for “three new types of food”—including forest resource protein foods. In the United States, edible insects and insect-based foods are subject to compliance with the Federal Food, Drug, and Cosmetic Act (FD&C Act) [90]; the European Union has extended the regulatory scope of the Novel Food Regulation (Regulation (EU) 2015/2283) to include all insect-derived products intended for human consumption (whole insects, insect parts, or extracts) [90]; and in the United Kingdom, insect-based enterprises are mandated to submit safety evidence dossiers to the Food Standards Agency (FSA) [91]. Driven by advancements in manufacturing technologies for related protein-based foods and the gradual refinement of regulatory systems, a growing number of governments have begun conditionally authorizing the market entry of select forest resource protein foods (Table 1). For instance, Singapore became the first nation to grant approval for the commercial sale of cell-cultured chicken in 2020 [108]; the U.S. FDA has successively approved Upside Foods and Eat Just for the production and sale of cell-cultured chicken (Table 1); and Aleph Farms is pursuing regulatory approval for the sale of cell-cultured steak in the United Kingdom and Switzerland. Additionally, governments in Israel, Singapore, Canada, and the United States have permitted Israeli companies Remilk and Imagindairy to utilize fermentation-derived animal-free milk whey protein and casein in the manufacturing of various food products. Solar Foods’ air protein products have obtained novel food approval in Singapore, with plans to submit a Generally Recognized as Safe notification in the United States by 2025 and advance qualification approval with the European Food Safety Authority. In China, protein resources designated as “three new types of food” primarily encompass fermented proteins (e.g., milk basic protein, *Phylloporia ribis* fermented mycelium), algae (e.g., *Haematococcus pluvialis*, *Chlorella pyrenoidosa*, *Nostoc sphaeroides*, *Dunaliella salina*, *Euglena gracilis*, *Nannochloropsis* sp., *Chlamydomonas reinhardtii*), macrofungi (e.g., *Cordyceps cicadae* fruiting body, *Cordyceps militaris*), and insects (e.g., earthworm protein).

## 5. Conclusions

As a low-carbon and ecologically sustainable industry, the forest food sector holds strategic significance in addressing global food security challenges. Forest protein resources currently encompass insect, microbial, and plant proteins, as well as cell-cultured meat biofabricated from forest animal cells. Over the past decade, substantial breakthroughs have been achieved in the exploration, development, and utilization of forest protein resources, with certain protein products (e.g., plant-based proteins) having realized commercial-scale and industrial production. Notably, in the field of insect proteins, there are over 3000 edible insect species worldwide, with a market size reaching USD 3.2 billion in 2023 (projected to increase to USD 7.6 billion by 2028); companies like Ÿnsect in France have promoted the development of high-protein insects through genotyping chip technology. In plant-based proteins, the market output value of PBDA is projected to grow from USD 25.19 billion in 2022 to USD 69.8 billion by 2030. For microbial proteins, Finland’s Solar Foods has established the world’s first air-based protein production base, with a production capacity of 160 tons/year. In the cell-cultured meat sector, Singapore approved the sale of cell-cultured chicken meat in 2020, and companies such as Upside Foods in the United States are engaged in cell-cultured meat production. While ongoing technological innovations and discoveries will continue to drive forest protein resources and their processed foods to play a pivotal role in ensuring food security and mitigating environmental pressures, sustained multi-source financial investment remains imperative from an industrial perspective. To advance this field, it is essential to strengthen technological innovation and scientific research to enhance the quality of forest protein foods, reduce production costs, clarify the material basis for edibility, and address technical bottlenecks and management challenges at both scientific and industrial levels. From a product development standpoint, forest protein-based foods must transcend the “meat replication” paradigm and transition toward an innovative strategy centered on “forest characteristics first.” The focus should prioritize leveraging the inherent advantages of forest protein resources rather than merely mimicking conventional meat and dairy products.

To accelerate the industrial scaling-up process, two quantifiable short-term goals are proposed: first, in terms of resource exploration and standardization, complete nutritional component (protein content, amino acid score) and safety assessments for 50 forest insect proteins by 2025, and establish the first DIAAS (Digestible Indispensable Amino Acid Score) database for 10 insect species; second, in terms of cost control and capacity enhancement, achieve a 30% reduction in production costs of microbial fermentation proteins by 2026 (from the current approximately USD 200/kg to below USD 140/kg), and promote enterprises such as Solar Foods to increase the annual capacity of air-based protein from 160 tons to 500 tons.

## Figures and Tables

**Table 1:** Partial examples of alternative protein food start-ups.

Start-Ups	Product	Country	Year of Establishment	URL
INTAKE	Microalgae protein	Republic of Korea	2023	www.shopintake.com (accessed on 5 September 2024)
Remilk	Fermentation-derived animal-free whey protein	Israel	2019	www.remilk.com (accessed on 5 September 2024)
Imagindairy	Fermentation of animal-free whey protein and casein	Israel	2020	www.imagindairy.com (accessed on 5 September 2024)
Perfect Day	Fermentation-derived animal-free whey protein	America	2014	https://perfectday.com (accessed on 7 September 2024)
The Cultivated B	Cultivating proteins	Germany	2021	https://www.thecultivatedb.com (accessed on 8 September 2024)
Solar Foods	Carbon dioxide and hydrogen replace sugar for fermenting microbial protein	Finland	2017	https://solarfoods.com (accessed on 14 September 2024)
Air Protein	Carbon dioxide and hydrogen replace sugar to ferment microbial protein	America	2019	https://www.airprotein.com (accessed on 17 September 2024)
Eden Brew	Non-dairy milk	Australia	2021	https://www.edenbrew.com.au (accessed on 17 September 2024)
Meati Foods	Plant-based steak produced through fungal fermentation	America	2016	https://meati.com (accessed on 17 September 2024)
Pan’s Mushroom Jerky	Mushroom meat	Malaysia	2008	https://jerkybrands.com (accessed on 19 September 2024)
Moku	Plant-based beef jerky Mushroom meat	America	2019	https://mokufoods.com (accessed on 19 September 2024)
Moving Mountains	Plant-based meatballs *Pleurotus eryngii* meat	Britain	2016	https://movingmountainsfoods.com (accessed on 23 September 2024)
Shroomeats	Mushroom pie Mushroom meatballs	America	2018	https://www.shroomeats.co (accessed on 25 September 2024)
Green Monday	Mushroom meat	China	2012	https://greenmonday.org (accessed on 27 September 2024)
MeatTheEnd	Alternative meat protein ingredients	Israel	2020	https://www.meattheend.tech (accessed on 15 December 2024)
Eatkind	Insect protein food	India	2021	https://www.eatkind.co (accessed on 15 December 2024)
Ÿnsect	Insect Feed and Fertilizer Insect Food Ingredients	France	2011	https://www.ynsect.com (accessed on 16 December 2024)
Hey Planet	Insect protein bars Insect snacks Insect cooking ingredients	Denmark	2016	https://www.hey-planet.com (accessed on 16 December 2024)
Beta Hatch	Insect Feed and Fertilizer Insect Food Ingredients	America	2015	https://betahatch.com (accessed on 17 December 2024)
Mighty Cricket	Insect protein powder Insect protein oatmeal	America	2018	https://mightycricket.com (accessed on 17 December 2024)
Protirax	Edible insect farming	Netherlands	2009	https://protix.eu (accessed on 17 December 2024)
Aspire Food	Edible insect farming	Britain	2018	https://aspirefg.com (accessed on 17 December 2024)
The Bug Factory	Edible insect farming	Britain	2019	https://bugfactory.co.uk (accessed on 19 December 2024)
Oshi	Plant-based salmon meat	Israel		https://oshi.com (accessed on 19 December 2024)
Lypid	Plant-based fat	America	2020	https://www.lypid.co/ (accessed on 19 December 2024)
Revo Foods	Plant-based salmon Mushroom-based salmon	Austria	2020	https://revo-foods.com (accessed on 5 November 2024)
Mycorena	Fungal protein	Sweden	2017	https://mycorena.com (accessed on 5 November 2024)
Redefine Meat	Plant-based burger	Britain	2018	https://www.redefinemeat.com/uk (accessed on 17 November 2024)
Smile Organic	Plant-based milk alternatives	Canada	2019	https://smileorganic.com (accessed on 13 November 2024)
Impossible Food	Plant-based chicken nuggets, sausages, ground beef, and pork	America	2011	https://impossiblefoods.com (accessed on 23 November 2024)
The Plantly Butchers	Plant-based sausage Plant-based bacon	Germany	2020	https://the-plantly-butchers.com/en (accessed on 21 November 2024)
Impossible	Plant-based chicken, Cultured meat burger	America	2011	https://impossiblefoods.com (accessed on 18 November 2024)
Foods Rebellyous	Plant-based chicken, Cultured meat burger	America	2017	https://www.rebellyous.com (accessed on 5 November 2024)
Foods Beyond Meat	Plant-based chicken, Cultured meat burger	America	2009	https://www.beyondmeat.com (accessed on 9 November 2024)
Joes Future Food	Cell-cultured pork Cell-cultured pig fat	China	2019	http://www.joesfuturefood.com (accessed on 1 November 2024)
Mosa Meat	Cell-cultured meat	Netherlands	2013	www.mosameat.com (accessed on 25 November 2024)
Mooji Meats	3D printed cell-cultured meat	Britain	2019	www.moojimeats.com (accessed on 17 November 2024)
Upside Food	Cell-cultured chicken meat	America	2015	https://upsidefoods.com (accessed on 11 November 2024)
GoodMeat/Eat Just	Cell-cultured chicken meat	America	2016	https://www.goodmeat.co (accessed on 13 November 2024)
Aleph Farms	Cell-cultured steak	Israel	2017	https://aleph-farms.com (accessed on 11 November 2024)
Meatable	Cell-cultured meat hamburger	Netherlands	2018	https://meatable.com (accessed on 12 November 2024)
Wildtype	Cell-cultured salmon	America	2016	https://www.wildtypefoods.com (accessed on 15 November 2024)
Avant Meat	Cell-cultured fish meat	China	2018	https://www.avantmeats.com (accessed on 15 November 2024)
Bluu Seafood	Cell-cultured fish meat	Germany	2020	https://www.bluu.bio (accessed on 15 November 2024)
Vow	Cell-culture quail meat	Australia	2019	https://www.vowfood.com (accessed on 5 November 2024)
Gourmey	Cell-culture foie gras	France	2019	https://www.gourmey.com (accessed on 16 November 2024)
Ivy Farm Technologies	Cell-cultured meat	Britain	2022	https://www.ivy.farm/ (accessed on 25 November 2024)
MeatAfora	Cell-cultured beef	Israel	2021	https://meatafora.com (accessed on 17 November 2024)
SciFi Foods	Cell-cultured beef	America	2019	https://scififoods.com (accessed on 16 November 2024)
Aleph Farms	Cell-cultured meat	Israel	2017	www.aleph-farms.com (accessed on 15 November 2024)

Note: Part of the data comes from reference [30], and part comes from web search (www.foodtalks.cn).

## Data Availability

No new data were created or analyzed in this study. Date Sharing is not applicable.
